# Gastric ischemic conditioning before esophagectomy: contemporary practices and insights from an international survey

**DOI:** 10.1007/s00464-026-12725-5

**Published:** 2026-03-16

**Authors:** Alberto Aiolfi, Davide Bona, Andrea Sozzi, Yves Borbély, Luigi Bonavina, G. I. C. Collaborative Group, Ahmed Abdelsamad, Alan Patrick Ainsworth, Jacopo Andreuccetti, Filippo Ascari, Karim Ataya, Gian Luca Baiocchi, Andrea Balla, Hasan Batirel, Maria Bencivenga, Adrian Billeter, Antonio Biondi, Damien Bouriez, Giuseppe Brisinda, Andrea Celotti, Giovanni Cestaro, Yin-Kai Chao, Edward Cheong, Mircea Chirica, Prokopis Christodoulou, Luca Cigagna, Davide Citterio, Xavier Benoit D‘Journo, Andrew Davies, Roberto De Anton, Andrew De Beaux, Pieter De Heer, Dionysios Dellaportas, Lieven Depypere, Jessie Elliott, Moustafa Elshafei, Alessia Fassari, Agostino Fernicola, Lorenzo Ferri, Uberto Fumagalli Romario, Giovanni Maria Garbarino, Suzanne Gisbertz, Ioannis Gkoutziotis, Ines Gockel, Antonietta Gerarda Gravina, Tristan Greilsamer, Ewen Griffiths, Caroline Gronnier, Christian Gutschow, Osman Serhat Güner, Nader Hanna, Jakob Hedberg, Nienhüser Henrik, Petre Hoara, Arnulf Hölscher, Nidal Iflazoğlu, Takeharu Imai, Orestis Ioannidis, Jan Johansson, Aristotelis Kechagias, Ebrahimi Keramatollah, Fredrik Klevebro, Efstathioa Kotidis, Paul Leeder, Xuefeng Leng, John Lipham, Donald Low, Sheraz Markar, Javier Martínez Caballero, Kristel Mils, Fernando Mingol Navarro, Luyer Misha, Daniela Molena, Stefan Mönig, Philippe Nafteux, Grard Nieuwenhuijzen, Magnus Nilsson, Fabio Massimo Oddi, Akihiko Okamura, Felipe Carlos Parreño-Manchado, Raffaele Pellegrino, Kyle Perry, Alexander Phillips, Gaetano Piccolo, Guillaume Piessen, Calin Popa, Dario Potkonjak, Daniel Reim, Elisa Reitano, Riccardo Rosati, Ioannis Rouvelas, Carlo Alberto Schena, Lars Schiffmann, Dimitrios Schizas, Francisco Schlottmann, Thomas Schmidt, Marcel Schneider, Sebastian Schoppmann, Rivfka Shenoy, Wolfgang Schroeder, Aleksandar Simic, Ognjan Skrobic, Vladimir Sljukic, Jennifer Straatman, Dimitrios Theodorou, Tania Triantafyllou, Mehmet Akif Üstüner, Mustafa Yener Uzunoglu, Gijs Van Boxel, Elke Van Daele, Hanne Vanommeslaeghe, Dejan Velickovic, Neil Welch, Omer Yalkin, Jörg Zehetner, Maurizio Zizzo

**Affiliations:** 1 I.R.C.C.S. Ospedale Galeazzi – Sant’Ambrogio, Division of General Surgery, Department of Biomedical Science for Health, University of Milan, Via C. Belgioioso, 173, 20157 Milan, Italy; 2 Department of Visceral Surgery and Medicine, Inselspital, Bern University Hospital, Bern, Switzerland; 3 Department of Pharmacy, Health and Nutrition Sciences, Azienda Ospedaliera di Cosenza, Division of General and Foregut Surgery, University of Calabria, Cosenza, Italy

**Keywords:** Esophagectomy, Ischemic conditioning, Laparoscopy, Embolization, Survey

## Abstract

**Background:**

Gastric ischemic conditioning (GIC) before esophagectomy has been proposed to enhance the vascular submucosal network of the gastric conduit and perfusion at the anastomotic site. Its advantages remain controversial due to inconsistent literature findings, often attributed to heterogeneity in patient selection, targeted vessels, timing, and variations in GIC technique. We conducted a survey to assess contemporary surgical practices regarding GIC utilization prior to esophagectomy among expert foregut surgeons.

**Methods:**

A Google-based survey was conducted in accordance with the CHERRIES checklist, developed following an extensive literature review and directed towards expert foregut surgeons. The survey comprised 39 questions covering demographic data, professional experience, surgical modalities for esophagectomy, indications, timing, and technical aspects for GIC.

**Results:**

Overall, 115 expert foregut surgeons participated in the survey (response rate 76.7%). Overall, 56.4% indicated that they do not perform GIC whereas 43.6% reported utilizing GIC before esophagectomy. Main reasons for not performing GIC included lack of supporting literature (57.1%) and no clear benefit in reducing AL rate (42.9%). Selective GIC use was most often based on celiac trunk stenosis or calcification (67.7%), history of coronary stenting/bypass (48.4%), and thoracic aorta calcification (41.9%). Overall, 59.6% of experts using GIC preferred laparoscopy while 40.4% favored embolization. Laparoscopy was preferred for cancer staging, jejunostomy formation, and hospital availability; embolization was preferred for its simplicity, avoidance of general anesthesia, absence of adhesions, and ability to dynamically assess the vascular anatomy intraprocedural. The left gastric artery was the most frequently targeted vessel (> 90%) for both laparoscopy and embolization, either individually or in combination with the short gastric vessels or the left gastroepiploic artery. Almost 70% of GIC users indicated a preference for performing GIC ≥ 14 days before esophagectomy.

**Conclusions:**

The survey indicates that less than half of the experts support GIC prior to esophagectomy, preferring its selective application. Laparoscopy is preferred over embolization, likely due to better tumor staging and greater hospital availability. Most respondents also prefer GIC to be performed more than 14 days before esophagectomy.

**Supplementary Information:**

The online version contains supplementary material available at 10.1007/s00464-026-12725-5.

In the context of multimodal therapy, esophagectomy with lymphadenectomy and reconstruction of digestive continuity using a gastric conduit is considered the gold standard surgical treatment for esophageal cancer [1–6]. Anastomotic leak (AL) remains a significant complication, affecting up to 30% of patients, and is associated with increased mortality, prolonged hospitalization, delayed initiation of oral intake, risk of reintervention, higher recurrence rates, and reduced overall and disease-free survival [6–9]. Despite being multifactorial, ischemia at the anastomotic site is regarded as a key contributor to AL development, prompting investigation into various perioperative strategies to mitigate this issue [10, 11]. Gastric ischemic conditioning (GIC) through partial devascularization of the upper stomach before esophagectomy was first described in 1995 to enhance adaptation of the vascular submucosal network through partial devascularization of the upper stomach, thereby improving perfusion at the future anastomotic site [12]. Nonetheless, the potential benefits of GIC prior to esophagectomy remain subject to debate, with some studies and meta-analyses reporting a significant decrease in AL rates, and others failing to confirm these results [13–25]. This may be in part due to the heterogeneity in patient selection, choice of vessels for ligation, timing from GIC to esophagectomy, and preferred technique (angioembolization versus laparoscopy) [26]. In light of ongoing uncertainty, we conducted a survey to evaluate current surgical practices among expert foregut surgeons and key factors influencing decision-making regarding the implementation of GIC prior to esophagectomy.

## Materials and methods

The present cross-sectional study was conducted according to the ethical guidelines for good research and practice published by the World Health Organization [27] and to the E-Surveys Checklist for Reporting Results of Internet (CHERRIES) [28]. The primary objective of the survey was descriptive, focusing on contemporary practices related to GIC utilization.

This is a google-based survey targeted at experts’ upper gastrointestinal surgeons from different hospital settings and countries. The questionnaire was conducted in English and based on 39 questions divided into demographic data, personal/institutional experience, and preferred surgical approach to esophagectomy (questions 1–13). GIC utilization, criteria for utilization, timing of GIC before esophagectomy (questions 14–26), and technical details of both laparoscopic and angioembolization methods were also collected (questions 27–39) (Online appendix 1).

The survey was created through the collaboration of four expert surgeons (AA, DB, YB, and LB) following a comprehensive review of relevant literature. The survey was critically reviewed by a select panel of international members of the European Foregut Society (EFS) who approved the final version of the survey prior to distribution. The internal consistency of the survey for evaluating coherence items was assessed using Cronbach’s alpha, which yielded a value of 0.87 thus suggesting good reliability [29]. Participation in the survey was entirely voluntary, and no incentives were provided. All participants received detailed information regarding the survey’s objectives, methodology, data to be collected, and anticipated benefits. Respondents had the opportunity to review and amend their responses after viewing all questions. To safeguard against unauthorized access, all data were stored on a dedicated, password-protected computer accessible only to the principal investigator (AA). Informed consent was obtained prior to survey completion. To ensure the verification of unique site visitors, respondents’ IP addresses were recorded, and each user was restricted from submitting more than once upon completion of the form.

Completion of all responses to the questionnaire was mandatory. The estimated time required to complete the questionnaire was approximately 15 min. The questionnaire was sent to referred experts from December 1st 2024 through June 1st 2025, and three calls for participation were launched within this period. Survey items included multiple-choice and dichotomous (yes or no) questions. There were no free-text questions. All data were analyzed anonymously.

Participants were enlisted through targeted email correspondence and outreach via LinkedIn profiles. The electronic questionnaire was pre-tested for functionality and distributed using Google Forms (Google LLC, Mountain View, CA, USA). The survey was endorsed and disseminated with the support of the European Foregut Society (EFS).

The data analysis was performed using descriptive statistics, presenting the percentage of agreement and median (IQR) using SPSS version 26.0. No Institutional Review Board (IRB) approval was necessary for this study since data were de-identified, analyzed anonymously without patient-related data.

## Results

**Fig. 1:**
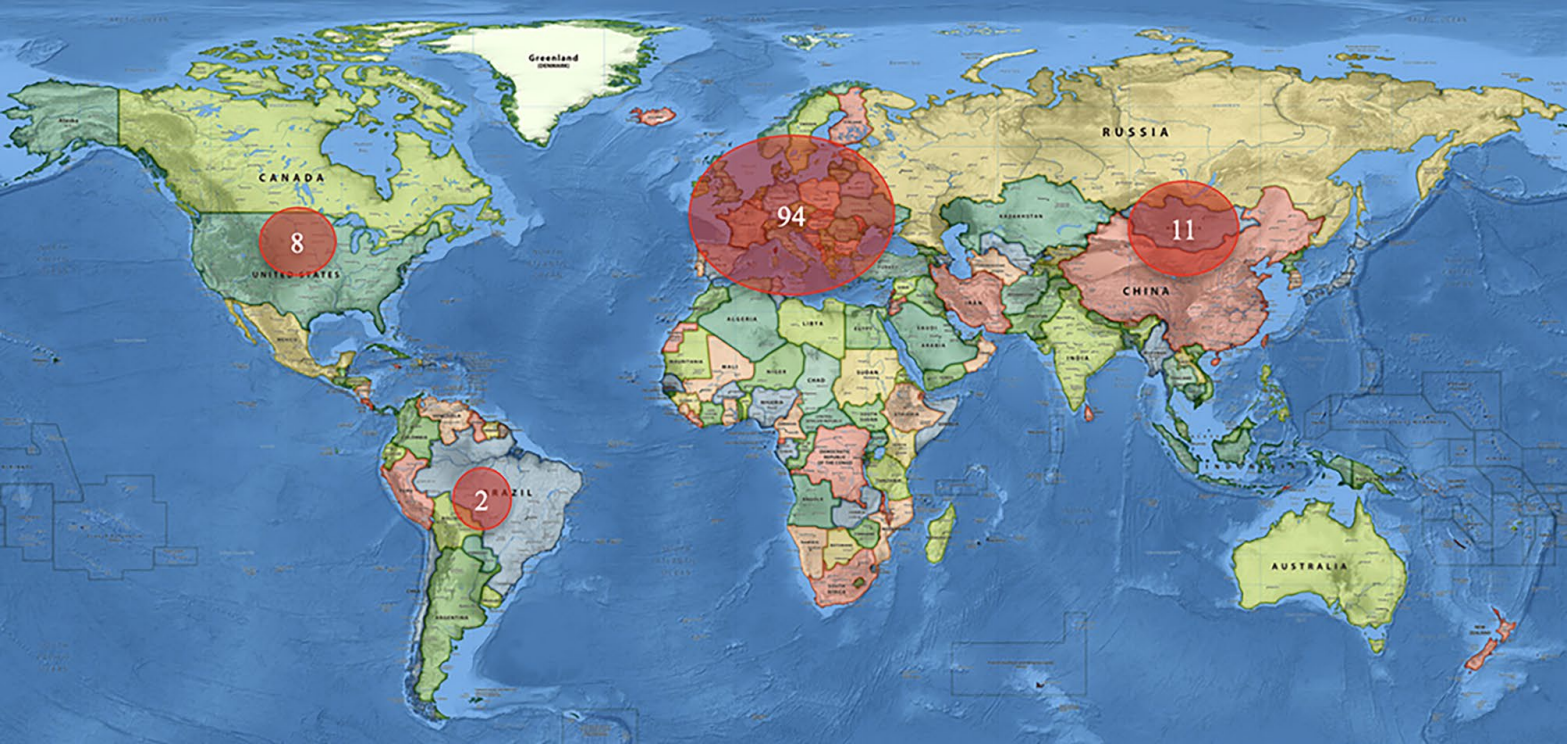
Worldwide distribution of the survey participants

One hundred fifty expert upper-GI surgeons were invited via personal email invitation. The list of experts was drafted based on experience in esophageal surgery and/or recently published research on GIC before esophagectomy. In total, 115 worldwide expert surgeons participated in this one-round survey (response rate 76.7%). The median age of respondents was 45 years, ranging from 28 to 70, with the majority being males (85.5%). Most of the participants were from Europe (80.9%) (Fig. 1). In terms of work environments, 54.8% of surgeons were from dedicated upper GI units, 32.2% from general surgery units, and 9.6% from thoracic surgery units. The majority of surveyed surgeons belonged to academic institutions (86.1%). Among the participants, 69.2% were consultant surgeons, 28.9% were chief of department, and 1.9% were fellows. Minimally invasive (45.2%), robotic (24.3%), hybrid (21.7%), and open (8.7%) transthoracic esophagectomy were the most commonly adopted approaches. The median number of esophagectomies per center was 40 procedures/year (range 15–650).

In summary, 56.4% of respondents indicated that they do not perform GIC prior to esophagectomy, whereas 43.6% reported utilizing GIC. Reasons for not performing GIC before esophagectomy were lack of evidence from the literature (57.1%), no perceived benefits on decreasing AL rate (42.9%), increased costs (17.5%), and increased GIC-related and post-esophagectomy complications (12.7%). Among surgeons performing GIC, the most commonly reported criteria for selective GIC application were presence of celiac trunk stenosis/calcification (modified NASCET score) (67.7%), previous coronary stenting/bypass (48.4%), thoracic aorta calcification (UCS score) (41.9%), planned cervical location of the anastomosis (35.5%), active smoking status (32%), diabetes (29%), and previous cerebrovascular accident (22.6%) (Table 1).

**Table 1:** Preferred preoperative criteria for selective GIC utilization

Celiac trunk stenosis/calcification (modified NASCET score)	67.7%
Previous coronary stenting/bypass	48.4%
Thoracic aorta calcification (UCS score)	41.9%
Location of the anastomosis (cervical)	35.5%
Active smoker	32.3%
Diabetes	29%
Cerebrovascular disease	22.6%
Neoadjuvant therapy	16.1%
Age > 65 yrs	16.1%

The timing of GIC varied according to the necessity for neoadjuvant (NA) treatment. The majority (73.3%) of respondents indicated a preference for performing GIC ≥ 14 days prior to esophagectomy in patients not requiring NA therapy. For patients requiring NA treatment, 44.9% preferred to conduct GIC before initiating NA therapy, often in conjunction with jejunostomy placement for those experiencing nutritional deficiency or significant weight loss. Following completion of NA therapy, 51% of respondents favored performing GIC between the 4th and 6th week after chemotherapy, 14.3% preferred the 6th to 8th week, and 4.1% opted for more than 8 weeks post-NA treatment. Additionally, in patients undergoing NA therapy, 72.6% of respondents preferred GIC within 14 days before esophagectomy.

Laparoscopy was selected by 59.6% of respondents as the preferred approach. The left gastric artery was the most frequently ligated vessel (90.3%), either individually or in combination with the short gastric vessels (57.1%) or the left gastroepiploic artery (17.9%). The right gastric artery was considered for ligation by 10.7% of participants. In terms of vessel closure techniques, 46.4% utilized a combination of clips and energy devices, while others reported exclusive use of Hem-o-loc clips (35.7%), energy devices (7.1%), or staplers (7.1%). Most practitioners favored vessel closure at the origin, whereas 25% performed closure near the stomach. The majority of respondents (78.6%) did not perform lymphadenectomy around the celiac axis during GIC. Notably, during GIC, 42.9% of surgeons considered performing a jejunostomy, 10.7% opted for immediate gastric tubulization, and 39.3% performed intraoperative indocyanine green (ICG) fluorescence assessment of the gastric conduit. There were cases of postoperative bleeding necessitating reoperation, though this did not delay the timing of esophagectomy. Increased adhesions, greater risk of bleeding, and difficult lymphadenectomy at the time of esophagectomy were reported when preemptive devascularization was performed at the origin of the left gastric vessel. Overall, laparoscopic GIC was favored over embolization due to the opportunity for staging laparoscopy or jejunostomy formation, perceived enhanced precision, and greater availability within hospitals. Embolization was identified as the preferred GIC technique by 40.4% of respondents. The left gastric artery was the most frequently embolized vessel (94%) alone or in combination with short gastric vessels (36.8%) or left gastroepiploic artery (36.8%). The right gastric artery was considered for embolization by 26.3% of respondents. Coiling was the favored method for angioembolization (63.2%), with a combination of coils and glue preferred by 31.6% of respondents. Reported complications related to angioembolization included pancreatitis and partial splenic infarction. The primary reasons for preferring embolization were its simplicity and minimally invasive nature, absence of peritoneal adhesions, lack of requirement for general anesthesia, and the ability to assess vascular anatomy and dynamic flow intra-procedurally.

The majority of participants utilizing GIC indicated a perceived effect of GIC in reducing both the rate (80.9%) and severity (78.7%) of post-esophagectomy AL. Additionally, 63.8% reported that GIC may contribute to lowering the incidence of AS, while 72.3% believed that GIC could potentially reduce GC necrosis during esophagectomy.

## Discussion

The use of GIC prior to esophagectomy remains a topic of debate. Based on the findings from our survey, less than half of the respondents reported considering GIC before esophagectomy; only 15% indicated support for its routine use, while 30% favored selective application in high-risk patients. Angioembolization and laparoscopy are both employed in current practice; however, laparoscopy appears to be generally favored due to its suitability for concomitant diagnostic assessment, feeding jejunostomy placement, and wider availability of hospital resources. The left gastric artery is usually the targeted vessel, either alone or in combination with the short gastric and/or left gastroepiploic artery. The majority of respondents indicated a preference for performing GIC more than 14 days prior to esophagectomy, irrespective of whether neoadjuvant therapy was administered.

The esophagogastric anastomosis represents one of the most technically demanding parts of esophagectomy. Multiple factors including longitudinal tension, operative technique, malnutrition, patient comorbidities, and insufficient blood supply to the gastric conduit may contribute to anastomotic failure [30, 31]. Accordingly, it is essential to optimize conduit perfusion and preserve an effective submucosal vascular network at the anastomotic site [32, 33]. Physiological gastric perfusion is sustained by large capillaries that are oriented perpendicular to both gastric curvatures, along with smaller branches running parallel to the curvatures, thereby forming a complex vascular network. The ligation of the left gastric artery, left gastroepiploic artery, and short gastric vessels implies a reorganization of this network [34–37]. As a result, perfusion within the gastric conduit is maintained primarily through the right gastroepiploic artery on the greater curvature, with the distal conduit depending on small, longitudinal collateral connections. Preoperative GIC has been introduced to facilitate vascular adaptation and enhance the submucosal vascular network. Improved vascular and oxygen supply at the tip of the gastric conduit and anastomotic site following GIC has been demonstrated via Doppler flowmetry, fluorescence microscopy, hyperspectral imaging, and angiography [16, 17]. Correspondingly, histological findings have revealed neoangiogenesis, increased microvessel density, and vessel hypertrophy [33, 38].

While no randomized trial has been published on this topic, previous studies and meta-analyses have shown encouraging outcomes for GIC prior to esophagectomy [22–26]. Nonetheless, its incorporation into routine surgical practice remains limited. In this context, our survey reveals that most respondents (56.4%) do not perform GIC, citing insufficient literature evidence, lack of perceived effect on AL, increased costs (17.5%), and concerns about GIC-related complications. Only a minority supported its routine utilization while almost 30% suggest a selective utilization in high-risk patients. Most of GIC users (67.7%) considered selective GIC utilization in patients with celiac trunk stenosis/calcification assessed through the modified NASCET score [39–41]. Additional factors include previous coronary stenting/bypass, thoracic aorta calcification assessed by uniform calcification score (UCS) [42, 43], cervical anastomosis, smoke, diabetes, and prior cerebrovascular accident. Artificial intelligence-driven, patient-specific algorithms could improve preoperative risk prediction for AL and help identify those who may benefit from GIC.

The optimal timing for GIC has yet to be clearly defined. Existing protocols typically recommend a period of 12–18 days, or approximately 2–3 weeks; however, some practices employ intervals exceeding 30 days [15, 24]. Currently, there are no standardized protocols, and within published series timing varies due to logistical challenges and operative scheduling. Experimental research has measured circulation at the gastric fundus and proximal stomach following GIC within periods ranging from 1 to 90 days [34, 44]. These studies demonstrate that after an initial decrease, perfusion gradually returns to baseline levels over 2 to 3 weeks post-ligation. Veeramootoo et al. prospectively evaluated the impact of surgical timing after ischemic conditioning in a cohort of 42 patients and concluded that performing esophagectomy two weeks after GIC significantly reduced AL rates and conduit failure (P < 0.0001) [14]. In contrast, other studies failed to report a significant impact of GIC timing [35, 37]. In our study, about 73% preferred an interval of over 14 days from GIC to esophagectomy for patients without NA treatment. For those given NA therapy, most respondents favored scheduling GIC 4–6 weeks after NA completion and waiting more than 14 days before esophagectomy (72.6%). This preference may provide a balance between improved conduit neovascularization and reduced peritoneal adhesion.

Laparoscopy was identified as the preferred GIC technique by 59.6%. Justifications encompassed the potential for staging laparoscopy or jejunostomy creation, perceived superior precision, and higher hospital availability. Conversely, angioembolization was preferred by 40.4% of respondents. The rationale included procedural simplicity, minimally invasive approach, absence of peritoneal adhesions, avoidance of general anesthesia, and the capacity for intraprocedural vascular anatomy and dynamic flow assessment. However, the selection of the preferred GIC technique might also be influenced by hospital resources, potential complications, and patient anatomy. Although our survey was not specifically designed to address this aspect, previous studies reported splenic infarction and pancreatitis following embolization, whereas an increased risk of bleeding and peritoneal adhesions occurred after laparoscopic GIC [45–53]. Laparoscopic GIC is an extra surgical procedure with additional costs, the potential for complications (i.e., bleeding or conversion to open), requires general anesthesia, and may lead to more difficult lymphadenectomy due to adhesions and scarring at vessel sealing sites, especially with stapler use. Finally, laparoscopic GIC might be difficult in challenging situations such as patients with large hiatus hernia and cranial displacement of the left gastric artery pedicle and in obese patients. Conversely, angioembolization might be performed under local anesthesia with radial or femoral artery access. Despite its minimally invasive nature, this technique incurs elevated costs associated with specialized equipment and materials. Additionally, embolization may be partial or unsuccessful due to technical challenges, including vascular tortuosity particularly at the level of the splenic artery where the short-gastric vessels emerge [24–26].

The selection of arteries for closure/ligation might have a substantial impact on GIC outcomes; however, current literature is heterogeneous and lacks definitive conclusions. Furthermore, intraoperative surgical planning may need to be adjusted according to variations of vascular anatomy (i.e., hepatic accessory artery), paleness of the gastric conduit during GIC, or ICG findings. According to our survey, the left gastric artery was the preferred target for both angioembolization and laparoscopic GIC (94%), either alone or in combination with short gastric vessels (36.8%) or left gastroepiploic artery (36.8%). This is similar to previous studies [35–41]. Also, the technique for vascular closure/ligation might be different. In our survey, coils and glue were preferred for angioembolization, while a combination of Hem-o-loc clips and energy devices was preferred for laparoscopic GIC. The majority of clinicians favored vessel closure at the origin; however, approximately 25% prefer closure near the stomach. Employing Hem-o-loc or clips close to the gastric wall may reduce adhesions at the origin of the left gastric vessel, thereby potentially facilitating lymphadenectomy at stations 7, 8, and 9.

The current survey has strengths in considering the higher response rate compared to previous web-based surveys for healthcare professionals [54]. However, it has a few shortcomings that need to be addressed. First, it is important to note that the purpose of this survey was to descriptively report current GIC practices rather than to provide practice recommendations. Selection bias is a relevant consideration, as respondents were personally invited either due to their recognition as experts in the field or based on recent publications regarding GIC. This methodology may result in significant sampling bias due to the potential exclusion of other prominent foregut surgeons. Furthermore, up to 23% of invited experts did not respond to the invitation, which may have contributed to additional non-response bias. Second, all data were self-reported without independent verification, which may impact the accuracy and reliability of the results (reporting bias). Additionally, as the survey was administered only once, consistency measures such as the intraclass correlation coefficient were not assessed. Third, including respondents from multiple continents and countries introduces heterogeneity, given variations in surgical practices and hospital volumes; moreover, the predominance of European participants may restrict the generalizability of our findings. Fourth, the lack of a direct interview may result in questions being misinterpreted or answered inaccurately. Finally, because the survey did not address GIC-related complications or costs, robust data on these aspects were not collected. In accordance with the intentions of the respondents, a collaborative multicenter study is planned for the next future to shed light on the real effect, related complications and costs of GIC prior to esophagectomy.

## Conclusions

The survey findings suggest that less than half of the experts endorse the utilization of GIC prior to esophagectomy, with a preference for its selective application primarily in patients considered at high risk for AL. Both laparoscopy and embolization are reported as current practices; however, laparoscopy appears to be generally favored, possibly due to its enhanced tumor staging capabilities and wider availability in hospital settings. Additionally, performing GIC more than 14 days prior to esophagectomy emerged as the preferred timing among respondents.

## Supplementary Information

Below is the link to the electronic supplementary material.

Supplementary file1 (DOCX 14 KB)

## Data Availability

All raw data are available if required.
