# Correction: Gastric ischemic conditioning before esophagectomy: contemporary practices and insights from an international survey

**DOI:** 10.1007/s00464-026-12797-3

**Published:** 2026-04-08

**Authors:** Alberto Aiolfi, Davide Bona, Andrea Sozzi, Yves Borbély, Luigi Bonavina, G. I. C. Collaborative Group, Ahmed Abdelsamad, Alan Patrick Ainsworth, Jacopo Andreuccetti, Filippo Ascari, Karim Ataya, Gian Luca Baiocchi, Andrea Balla, Hasan Batirel, Maria Bencivenga, Adrian Billeter, Antonio Biondi, Damien Bouriez, Giuseppe Brisinda, Andrea Celotti, Giovanni Cestaro, Yin-Kai Chao, Edward Cheong, Mircea Chirica, Prokopis Christodoulou, Luca Cigagna, Davide Citterio, Xavier Benoit D‘Journo, Andrew Davies, Roberto De Anton, Andrew De Beaux, Pieter De Heer, Dionysios Dellaportas, Lieven Depypere, Jessie Elliott, Moustafa Elshafei, Alessia Fassari, Agostino Fernicola, Lorenzo Ferri, Uberto Fumagalli Romario, Giovanni Maria Garbarino, Suzanne Gisbertz, Ioannis Gkoutziotis, Ines Gockel, Antonietta Gerarda Gravina, Tristan Greilsamer, Ewen Griffiths, Caroline Gronnier, Christian Gutschow, Osman Serhat Güner, Nader Hanna, Jakob Hedberg, Nienhüser Henrik, Petre Hoara, Arnulf Hölscher, Nidal Iflazoğlu, Takeharu Imai, Orestis Ioannidis, Jan Johansson, Aristotelis Kechagias, Ebrahimi Keramatollah, Fredrik Klevebro, Efstathioa Kotidis, Paul Leeder, Xuefeng Leng, John Lipham, Donald Low, Sheraz Markar, Javier Martínez Caballero, Kristel Mils, Fernando Mingol Navarro, Luyer Misha, Daniela Molena, Stefan Mönig, Philippe Nafteux, Grard Nieuwenhuijzen, Magnus Nilsson, Fabio Massimo Oddi, Akihiko Okamura, Felipe Carlos Parreño-Manchado, Raffaele Pellegrino, Kyle Perry, Alexander Phillips, Gaetano Piccolo, Guillaume Piessen, Calin Popa, Dario Potkonjak, Daniel Reim, Elisa Reitano, Riccardo Rosati, Ioannis Rouvelas, Carlo Alberto Schena, Lars Schiffmann, Dimitrios Schizas, Francisco Schlottmann, Thomas Schmidt, Marcel Schneider, Sebastian Schoppmann, Rivfka Shenoy, Wolfgang Schroeder, Aleksandar Simic, Ognjan Skrobic, Vladimir Sljukic, Jennifer Straatman, Dimitrios Theodorou, Tania Triantafyllou, Mehmet Akif Üstüner, Mustafa Yener Uzunoglu, Gijs Van Boxel, Elke Van Daele, Hanne Vanommeslaeghe, Dejan Velickovic, Neil Welch, Omer Yalkin, Jörg Zehetner, Maurizio Zizzo

**Affiliations:** 1 I.R.C.C.S. Ospedale Galeazzi – Sant’Ambrogio, Division of General Surgery, Department of Biomedical Science for Health, University of Milan, Via C. Belgioioso, 173, 20157 Milan, Italy; 2 Department of Visceral Surgery and Medicine, Inselspital, Bern University Hospital, Bern, Switzerland; 3 Department of Pharmacy, Health and Nutrition Sciences, Azienda Ospedaliera di Cosenza, Division of General and Foregut Surgery, University of Calabria, Cosenza, Italy

**Correction to: Surgical Endoscopy** 10.1007/s00464-026-12725-5

The original online version of this article was revised to include the members of the G.I.C. Collaborative Group as credited collaborators.

The original article has been corrected.

