# Supplementary material for: Gastric ischemic conditioning before esophagectomy: contemporary practices and insights from an international survey
Source: Surg Endosc. 2026 Mar 16;40(7):5542–9. doi: 10.1007/s00464-026-12725-5 (PMC13369694; doi:10.1007/s00464-026-12725-5)
Supplement: Supplementary file 1 — Supplementary file1 (DOCX 14 KB) [file 464_2026_12725_MOESM1_ESM.docx]

**APPENDIX 1**

**Gastric ischemic conditioning (GIC) before esophagectomy - International survey**

<https://docs.google.com/forms/d/e/1FAIpQLScKDcdnLubM_3nnqhZ3tS_2PUqxxK4L0NlNyM1PJERi6ncXKA/viewform?usp=header>
